# 

**Published:** 1893-03

**Authors:** 


CONTENTS

VOL. 40. No. 3.

| | PAGE. |
|---|---|
| Physical Necessities | 49 |
| Behind the Counter | 51 |
| Mound Builders | 53 |
| Town and Country | 55 |
| Diphtheria | 57 |
| Free or Forced Vaccination | 58 |
| Women in the Sick Room | 59 |
| Smoking by Boys | 61 |
| Uncomfortable People | 62 |
| Saved His Friends and Died | 63 |
| Tannin in Tea | 64 |
| Healthy Bed Clothing | 64 |
| A Pulpit Defence of the Stage | 65 |
| Abuse of Cocaine | 66 |
| Stranger Than Fiction | 67 |
| Miscellaneous | 68 |
| Literary | 71 |
| Brawn and Brain | 72 |

INDEX TO ADVERTISEMENTS OF HALF A PAGE AND OVER.

| | PAGE. |
|---|---|
| Dr. Jaeger's Sanitary Woolens | I |
| National Mutual Insurance Co. | II |
| The Press Claims Co. | III |
| The Rip Van Winkle Rocker | IV |
| Herba Vita | V |
| Hall's Journal Premiums | VII |
| Washington Improvement Co. | VIII |
| J. Fehr's Compound Talcum | IX |
| Herba Vita | X |
| James Vick's Sons | X |
| Royal Baking Powder | XI |
| Buffalo Lithia Water | XI |

